# Development of the SCK CEN reference datasets for spent fuel safeguards research and development

**DOI:** 10.1016/j.dib.2020.105462

**Published:** 2020-03-21

**Authors:** Riccardo Rossa, Alessandro Borella

**Affiliations:** Environment, Health and Safety Institute, SCK CEN Belgian Nuclear Research Centre, 2400 Mol, Belgium

**Keywords:** Spent fuel, Nuclear safeguards, ORIGEN-ARP, Neutron emission, Gamma-ray emission

## Abstract

A set of reference spent fuel libraries has been organized in datasets containing the material composition and radiation emission (i.e. neutrons and gamma-rays) of spent fuel. The data was obtained with computer simulations using the ORIGEN-ARP code, which is part of the SCALE 6.1 package.

Both fuel assembly geometries for Pressurized Water Reactors (PWR 17 × 17) and Boiling Water Reactors (BWR 8 × 8) are included in the datasets, and for each geometry both uranium oxide (UO_2_) and mixed oxide (MOX) fuel materials are considered. The datasets contain the information for spent fuel with a broad range of initial enrichment (UO_2_ fuel) or initial Pu content (MOX fuel), discharge burnup, and cooling time.

For each simulation the neutron spectra, divided into contributions from (α,n) reactions, spontaneous fission, and total neutron emission, as well as total gamma-ray spectra are included. The neutron emission from selected isotopes is also reported, divided in contributions from (α,n) reactions and spontaneous fissions.

The datasets are publicly available and are in a format that facilitates the further data extraction and processing for applications in the safeguards research & development related to spent fuel measurement and verification.

**Specifications table**

**Table:** 

Subject	Nuclear Energy and Engineering
Specific subject area	Nuclear safeguards
Type of data	Table
How data were acquired	The data was generated with the ORIGEN-ARP 6.1 computer code.
Data format	Raw
Parameters for data collection	Fuel assembly geometry: • Pressurized Water Reactor 17 × 17 (PWR 17 × 17) • Boiling Water Reactor 8 × 8 (BWR 8 × 8) Fuel material type: • Uranium oxide (UO_2_) • Mixed oxide (MOX) Initial enrichment (UO_2_): 1.5% to 6.0% in steps of 0.5% Initial Pu content (MOX): 4.0% to 10.0% in steps of 1.0% Discharge burnup (UO_2_): 5 GWd/t to 70 GWd/t in steps of 5 GWd/t Discharge burnup (MOX): 5 GWd/t to 60 GWd/t in steps of 5 GWd/t Cooling time: 20 values ranging from 1 day to 100 years •1, 3, 10, 30, 100, 300 days • 1, 2, 3, 4, 5, 6, 7, 8, 9, 10, 20, 30, 50, 100 years
Description of data collection	The data was extracted from the output files of the ORIGEN-ARP simulations.
Data source location	Mol, Belgium
Data accessibility	Public repository. Repository name: “SCK-CEN spent fuel libraries”, Mendeley Data, v1 Direct URL to data: http://dx.doi.org/10.17632/vk5hv33p24.1

## Value of the data

• The dataset contains an extensive collection of spent fuel assemblies with varying initial fissile material content, burnup, and cooling time. The dataset serves as repository for neutron and gamma-ray emissions for spent fuel assemblies with different irradiation histories.
• Different fuel geometries (i.e. PWR, BWR) and fuel types (i.e. UO_2_, MOX) covering most of the irradiation histories in power reactors are included in the dataset. Moreover, the data is offered in a format that facilitates further extraction and processing.
• The dataset is especially intended for researchers in the field of nuclear safeguards and aims at supporting the research and development activities related to the measurement and verification of spent nuclear fuel. For example, the dataset can be used to study the influence of irradiation history on the resulting neutron and gamma-ray emissions from spent fuel.

## 1 Data

The datasets presented in this article are an extension of the previous work [1], [2], [3], carried out at the Belgian nuclear research centre (SCK CEN) considering fuel assembly geometries of Pressurized Water Reactors (PWR) and Boiling Water Reactors (BWR). For each geometry both uranium oxide (UO_2_) and mixed oxide (MOX) fuel materials are included.

The main folder “SCK-CEN spent fuel libraries” contains:

• Folder “BWR”: results for BWR 8 × 8 fuel geometry
• Folder “PWR”: results for PWR 17 × 17 fuel geometry
• “OverviewCases.xlsx”: overview of simulations per fuel type (i.e. BWR, PWR), fuel material (i.e. UO_2_, MOX), initial enrichment (IE), burnup (BU), and cooling time (CT)
• “Summary.xlsx”: tables with neutron emission (divided into contributions from (α,n), spontaneous fission, and total neutron) or gamma-ray emission (total gamma-ray) for all cases. Separate sheets for fuel type and fuel material.

Each subfolder (“BWR” or “PWR”) contains two additional subfolders:

• Subfolder “LEU”: results for UO_2_ fuel. Additional subfolders based on fuel initial enrichment.
• Subfolder “MOX”: results for MOX fuel. Additional subfolders based on fuel initial Pu content.

Each final subfolder contains files with two extension types:

• “.out”: these are the output files from the ORIGEN-ARP simulations
• “.xlsx”: these are the Excel files containing several sheets:
  ○ “SAN”: neutron spectra due to (α,n) reactions
  ○ “SSF”: neutron spectra due to spontaneous fissions reactions
  ○ “STO”: total neutron spectra
  ○ “SGA”: total gamma-ray spectra
  ○ “IAN”: neutron emissions per isotope, due to (α,n) reactions
  ○ “ISF”: neutron emissions per isotope, due to spontaneous fissions reactions

In case of results with low-enriched uranium (LEU) fuel material the naming convention for the files is:

**L0XXXYYY**

• XXX are the three digits for the initial enrichment – IE (e.g. 3.5% —> 350)
• YYY are the three digits for the discharge burnup – BU (e.g. 20 GWd/tU —> 200)

In case of results with mixed oxide (MOX) fuel material the naming convention for the files is:

**M0ZZZYYY**

• ZZZ are the three digits for the initial Pu content (e.g. 4.0% —> 040)
• YYY are the three digits for the discharge burnup (e.g. 20 GWd/tU —> 200)

## 2 Experimental design, materials, and methods

The ORGIEN-ARP 6.1 (Oak Ridge Isotope GENeration - Automatic Rapid Processing) [4] computer code was used to simulate the irradiation of fuel assemblies in nuclear power reactors and to specify the irradiation history of each case. The ORIGEN-ARP code was part of the SCALE (Standardized Computer Analyses for Licensing Evaluation) package until the version 6.1 [5].

A graphical user interface (GUI) is available for the setup of the simulation, and the GUI is composed of several panels.

### 2.1 First GUI panel: Express

The first panel (“Express”) allows the user to select the fuel material type (i.e. UO_2_ or MOX) and the fuel assembly geometry.

In the case of UO_2_ fuel the “w17 × 17” option was chosen to replicate the PWR 17 × 17 fuel geometry, whereas the “abb8 × 8–1” option was selected as example of the BWR 8 × 8 fuel geometry. In the case of MOX fuel the “mox17 × 17” or the “mox 8 × 8” cases were chosen for the PWR and BWR simulations, respectively.

In the case of UO_2_ fuel, the first panel allows also to select the total uranium content in the fuel and the initial enrichment. In the case of MOX fuel, the total heavy metal content, the combined percentage of Pu and Am over the heavy metal content (%(Pu + Am) / Heavy metal), and the percentage of Am over the combined Pu and Am content (%Am /%(Pu + Am)) can be specified.

In addition, the user in the first panel can specify the discharge burnup, the number of irradiation cycles, the number of libraries to be used per cycle, the cooling time, and the average power.

As summary, for the SCK CEN spent fuel libraries the following values were used:

• Fuel type: depending on the simulation
• Uranium: 1 ton (only for UO_2_ fuel)
• Heavy metal: 1 ton (only for MOX fuel)
• Enrichment: depending on the simulation (only for UO_2_ fuel)
• %(Pu + Am) / Heavy metal: depending on the simulation (only for MOX fuel)
• %Am /%(Pu + Am): none (only for MOX fuel)
• Burnup: depending on the simulation
• Cycles: depending on the simulation
• Libraries: 1 per cycle
• Cooling time: specified in the following panels
• Moderator density: default values proposed by ORIGEN-ARP (i.e. 0.723 g/cc for UO_2_ fuel, 0.72 g/cc for MOX fuel)
• Average power: 40 MW/MTU (for UO_2_ fuel) or 40 MW/MTHM (for MOX fuel)

### 2.2 Second GUI panel: Composition

The second GUI panel allows the user to specify the fuel initial composition. The default values proposed by ORIGEN-ARP were kept for the U and Pu isotopes, but oxygen was added to the list of isotopes in the fresh fuel in order to model the oxide forms. The natural isotopic composition was selected with a concentration of 134,500 g/t for UO_2_ fuel, or 134,421.15 g/t for MOX fuel.

The Pu isotopic vector used in all simulations with MOX fuel was taken from the Organization for Economic Co-operation and Development (OECD) MOX benchmark that is included in the ORIGEN-ARP manual as sample problem 6 [4]:

### 2.3 Third GUI panel: Neutron

In the panel “Neutron” the user can select the energy group structure used to calculate the neutron source spectra. The “238GrpSCALE” was the option chosen in the calculations, and this group structure extends from 10^−5^ eV up to 20 MeV. It is worth to mention that this panel determines only the division of the energy groups and is not related to the nuclear data library used by the code.

### 2.4 Fourth GUI panel: Gamma

Similar to the previous panel, the “Gamma” section of the GUI allows the user to select the energy group structure to calculate the gamma-ray spectra. For all simulations a 74-group library defined by the user was chosen. The group structure extends from 10^4^ eV up to 10 MeV.

### 2.5 Fifth GUI panel: Cases

The last GUI panel is also the most extensive one, since it allows the user to specify all the parameters for the irradiation cycles and decay calculations.

For each simulation in the SCK CEN spent fuel libraries one irradiation cycle lasted for maximum of 360 days, followed by 30 days of decay before starting the next irradiation cycle. The duration of the last irradiation cycle was adapted to reach the desired burnup level for the simulation. Following this approach, the fuel assembly is irradiated by a constant power level during all cycles, and a complete irradiation cycle leads to a burnup of 14.4 GWd/t.

After the last irradiation cycle, a decay calculation is performed to compute the fuel composition and corresponding neutron and gamma-ray emissions as a function of cooling time. A set of 20 cooling time values ranging from 1 day until 100 years were chosen to populate the database.

Table 1.

**Table 1:** Pu isotopic vector used in the simulations with MOX fuel. [4].

Pu isotope	% composition
Pu238	2.5
Pu239	54.7
Pu240	26.1
Pu241	9.5
Pu242	7.2
U234	0.00119
U235	0.25
U238	99.7488

Other options were selected in all ORIGEN-ARP simulations:

• no cut-off was set for the material composition so all isotopes are reported in the output file
• output precision was 6 digits for the values of the mass concentrations
• output precision was 4 digits for the neutron and gamma-ray emission
• the fuel matrix for the (α,n) evaluation was UO_2_
• bremsstrahlung was not considered in the model

## Declaration of Competing Interest

None.
